# Friction and Wear Behavior of Laser‐Induced Graphene Structures on Polyimide Films

**DOI:** 10.1002/smsc.202500335

**Published:** 2025-10-15

**Authors:** Milena Gleirscher, Stefan Zeiler, Paola Parlanti, Christine Bandl, Verena Maier‐Kiener, Francesco Greco, Sandra Schlögl

**Affiliations:** ^1^ Chemistry of Functional Polymers Polymer Competence Center Leoben GmbH Sauraugasse 1 8700 Leoben Austria; ^2^ Chair of Chemistry of Polymeric Materials Technical University of Leoben Franz Josef‐Straße 18 8700 Leoben Austria; ^3^ Department of Materials Science Montanuniversität Leoben Franz Josef‐Straße 18 8700 Leoben Austria; ^4^ Center for Materials Interfaces Istituto Italiano di Tecnologia viale R. Piaggio 34 56025 Pontedera Italy; ^5^ The Biorobotics Institute and Dept. of Excellence in Robotics & AI Scuola Superiore Sant’Anna viale R. Piaggio 34 56025 Pontedera Italy; ^6^ Interdisciplinary Center on Sustainability and Climate Scuola Superiore Sant’Anna Piazza Martiri della Libertà 33 56127 Pisa Italy

**Keywords:** direct laser writing, friction reduction, laser‐induced graphene, reciprocating ball‐on‐plate, surface texturing, tribology

## Abstract

Laser‐induced graphene (LIG) is formed by the conversion of certain carbon precursors when irradiated with a laser beam. Predesigned LIG patterns are scribed onto the precursor material in a low‐cost and maskless process, which enables the fabrication of flexible and electrically conductive materials for various applications. This study explores the friction and wear behavior of LIG from a polyimide precursor. Line patterns with different widths (200, 100, 50, and 30 μm) are introduced to modify the friction properties. An ultraviolet laser source with a nominal beam size of 2 μm is used, as it allows to scribe patterns with smaller dimensions and at higher resolution compared to the more commonly applied infrared laser sources. A distinct correlation is established between the pattern and its friction behavior, where lowering the line size results in a decrease in the coefficient of friction (COF). The wear behavior is evaluated, revealing gradual wear of the protruding LIG roughness peaks and a change in the graphenic material, which reduces the COF during the running‐in stage of the tribological testing. Due to its versatility in terms of precursor material, patterning options, and morphology modification, LIG represents a meaningful candidate for customized tribological applications.

## Graphical abstract

This study presents an investigation into the friction and wear behavior of laser‐induced graphene (LIG). Introducing line patterns of different sizes with this laser scribing approach results in a lowering of the coefficient of friction. The gradual wear of the protruding LIG patterns and the formation of wear debris are explored.

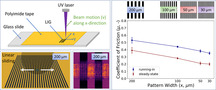

## 1 Introduction

The formation of graphene from commercial polymer films by laser‐induced pyrolysis has been extensively studied since the seminal work of Lin et al. in 2014.^[^ ^1^ ^]^ Among the various graphene fabrication techniques,^[^ ^2^ ^]^ transformative direct laser writing (DLW) has the advantage of a single‐step, fast, chemical‐free, and maskless conversion and patterning of the precursor using commercially available laser cutting/engraving equipment.^[^ ^3^, ^4^ ^]^

The formed 3D porous graphene films, referred to as laser‐induced graphene (LIG), are generated through a photochemically and thermally induced conversion of a precursor.^[^ ^5^ ^]^ Applicable precursor materials include high‐temperature engineering plastics, first and foremost polyimide (PI, Kapton),^[^ ^1^, ^6^, ^7^, ^8^ ^]^ though LIG formation on certain crosslinked vinyl polymers and epoxy resins has also been demonstrated.^[^ ^9^ ^]^ To date, increasing efforts are made to obtain LIG from bioderived materials.^[^ ^10^, ^11^ ^]^ In a more recent approach, inks or paints obtained from low molecular weight xanthene dyes are used as a new type of precursors for LIG formation and allow for LIG functionalization on virtually any surface.^[^ ^12^ ^]^

Properties of LIG include excellent thermal and electrical conductivity, good mechanical strength, and flexibility, paired with high porosity and specific surface area.^[^ ^13^ ^]^ These favorable characteristics combined with the possibility to (spatially) control the properties and the structural morphology of LIG by tuning the laser parameters and the available option of graphene functionalization^[^ ^8^, ^14^ ^]^ render LIG a popular area of research.^[^ ^15^ ^]^ Discussed shortcomings of LIG compared with conventionally fabricated graphene mainly mention a lack in quality and limitations in the selection of applicable precursor materials.^[^ ^16^, ^17^ ^]^ However, its overall comparatively straightforward and inexpensive production within a single synthesis or patterning step makes this method of graphene fabrication appealing for a wide range of applications,^[^ ^18^ ^]^ including optoelectronic devices, energy storage devices, biosensors, biomimetic devices, and sensorized soft robots.^[^ ^19^, ^20^, ^21^ ^]^ Recent advances in soft skin electronics, aimed at the implementation in intelligent healthcare, show a promising field for future LIG‐based applications.^[^ ^22^ ^]^ In addition, the utilization of LIG is highlighted in the fabrication of triboelectric nanogenerators (TENG), which are capable of converting mechanical motion (e.g., sliding) into electrical energy and, thus, harvest energy for autonomous devices.^[^ ^23^, ^24^, ^25^ ^]^

Graphene‐based materials possess excellent anti‐wear and friction‐reducing properties due to their self‐lubricating lamellar structure and film‐forming abilities, which qualifies them as potential candidates in tribological applications.^[^ ^26^, ^27^ ^]^ As a low‐cost and conveniently manufacturable graphene‐based material, LIG could be utilized in friction and wear related areas. However, to date, its macroscale lubrication performance is still underexplored. In 2022, Xue and coworkers^[^ ^28^ ^]^ reported on the potential of LIG as a solid lubricant. They were able to show good friction behavior of LIG fabricated from PI films on silicon substrates with varying surface roughness.

In addition to controlling the laser‐induced pyrolysis through a set of scribing parameters, DLW creates the opportunity to resolve high‐precision LIG patterns, thereby tuning the tribological characteristics of a polymer surface. In literature, the introduction of surface textures is a popular approach to change surface topography, surface roughness, and, consequently, the number of contact points, which is crucial for controlling the friction and wear behavior.^[^ ^29^ ^]^ The reduction of the real area of contact, lowering of the adhesion or the collection of wear debris are possible mechanisms that can contribute to improved tribological performance of structured surfaces.^[^ ^30^ ^]^ Etsion and coworkers^[^ ^31^, ^32^ ^]^ demonstrated the beneficial effects on the tribological performance of different mechanical components in the 90s, which promoted an ever‐increasing interest in the effects of surface texturing in different lubrication regimes.^[^ ^29^, ^33^ ^]^

Herein, we present an investigation into the arising opportunities from using LIG to obtain a conveniently patterned graphenic material and examine the resulting tribological properties. DLW using a UV laser source was used to write LIG structures onto a polyimide tape, a well‐established LIG precursor. Selected symmetrical line patterns with varying line widths and spacings ranging from 30 to 200 μm were fabricated, and the tribological behavior of the patterned surfaces was compared to those of a fully covered LIG surface. The obtained LIG morphology was assessed as a function of the laser processing parameters and characterized through optical and confocal microscopy, scanning electron microscopy (SEM) imaging, X‐ray photoelectron spectroscopy (XPS), and Raman spectroscopy. The tribological properties of LIG surfaces and the influence of surface patterning on LIG friction and wear were investigated, and a correlation between the sized line patterns and the evolution of the coefficient of friction (COF) could be established. Sliding‐induced changes to the LIG and the evolution of wear were examined by comparing the wear tracks formed by a variation in the number of measurement cycles.

## 2 Experimental Section

### 2.1 LIG Synthesis: Laser Scribing and Patterning

**Figure 1:**
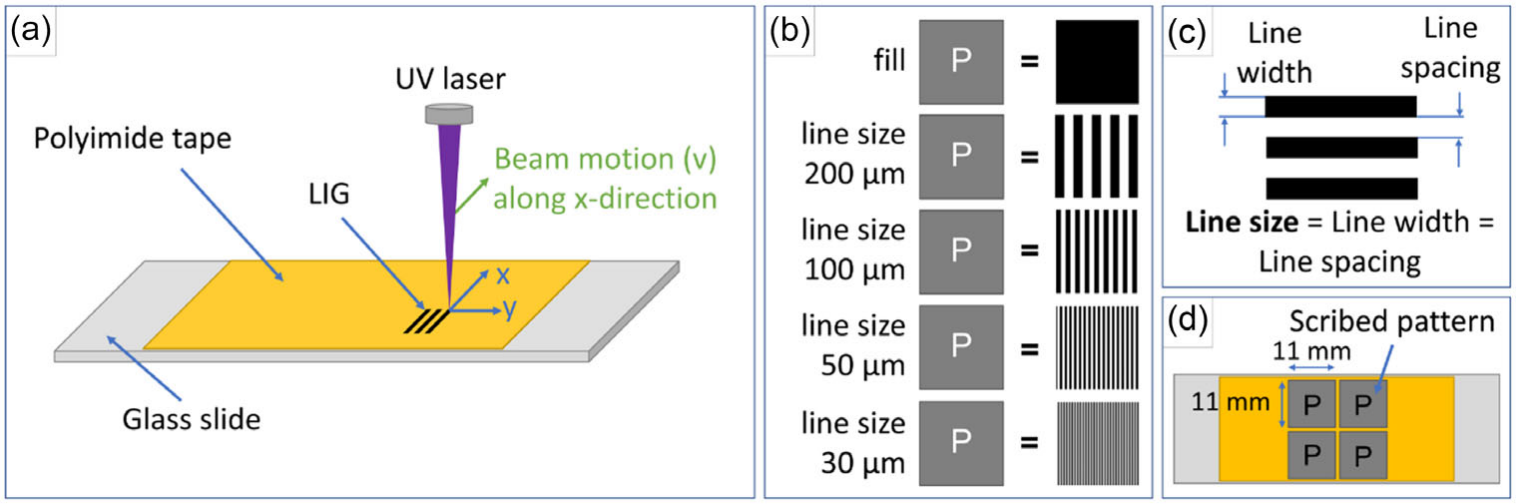
a) Schematics of the scribing of line patterns onto the PI tape with a UV laser source. b) Illustration of different pattern variations (fill and the various line sizes). c) Nomenclature of the geometric designation used to describe the fabricated line patterns. d) Schematics of a sample consisting of four individual patterns.

A laser cutter/engraver (Keyence 3‐Axis UV Laser Marker MD‐U1000C) operating with a YVO_4_ laser source at 355 nm wavelength, an output of 3 W/40 kHz, and a marking resolution of 2 μm was used to create LIG patterns on PI tape (Kapton HN, DuPont, 50 μm thickness with silicone glue, supplied by M&S Lehner GmbH) under ambient atmosphere. The PI tape was attached to glass microscopy slides with dimensions of 25 × 75 × 1 mm (ISO 8037/1, Espredia) for convenient sample handling. To exclude the impact of ambient humidity, the samples were stored in a desiccator. As shown schematically in **Figure** **1**a, the UV laser beam, with a power *P* of 15% and a pulse frequency of 210 kHz, was scanned across the polyimide film at a scan speed of 55 mm s^−1^ to write the individual line patterns. Five different patterns were fabricated, including four variations of differently sized line patterns (line width: 200, 100, 50, and 30 μm) and a fully covered LIG surface (fill) as displayed in Figure 1b. The line patterns all had equal line width and line spacing between the individual lines of the pattern (Figure 1c). Thus, the term “line size” is used in the following for brevity, defining both the width and the spacing. The smallest resolvable line size using the set laser parameters was 30 μm. Therefore, the individual lines in the 30 μm pattern were scribed in a single laser pass. All other patterns had to be scribed using more than one adjacent laser pass (a fill interval of 15 μm denotes the distance between the respective laser passes) to fill out the desired width of the thicker lines. The selected patterns were scribed in individual sized squares of 11 × 11 mm, and each sample consisted of four squares with individual patterns (Figure 1d).

### 2.2 LIG Characterization

#### 2.2.1 Optical Microscopy

The fabricated LIG patterns, LIG morphology, formed wear tracks, and the transfer films on the counterpart were investigated with a Stemi MAT 7 light microscope (Carl Zeiss GmbH) and its implemented Axiocam 208 color camera (Carl Zeiss GmbH) using bright and dark field mode, and a Hirox HR 5000(E) (Hirox Europe) digital optical microscope equipped with a High‐Range Motorized Triple Zoom Lens in bright field mode.

#### 2.2.2 Confocal Microscopy

The surface topography was characterized with a confocal microscope MicroProf(Fries Research & Technology GmbH). The measuring frequency was 320 Hz with a measuring speed of 365 μm s^−1^ and a lateral resolution of 1 μm. The measurement provides the intensity data of the collected light, which is used to determine the topography of the surface. The measurement data and 3D profiles were extracted from the intensity measurements with the associated software Mark III (Fries Research & Technology GmbH), and the corresponding height profiles were evaluated using a Python script.

#### 2.2.3 SEM

Focused Ion Beam Scanning Electron Microscope (FIB‐SEM) Helios Nano Lab 600i (Thermo Fisher Scientific Inc.) was used for SEM imaging. The images were acquired at 4 mm working distance, operating at 5 kV and 43 pA and using an Everhart–Thornley detector (ETD) collecting secondary electrons (SE).

#### 2.2.4 Raman Spectroscopy

Raman spectra were recorded with an alpha300 R Raman imaging microscope (WITec GmbH) with a laser wavelength ($\lambda_{l}$) of 532 nm. The laser power was set to 2 mW, and 50 single spectra, each with an integration time of 1 s, were accumulated. The spectra were obtained on the pristine LIG surface and on the wear tracks, formed by tribological testing, and averaged from 6 to 10 measurements to account for the localized nature of Raman measurements. The peak intensities of the D band ($I_{D}$), G band ($I_{G}$), and 2D band ($I_{\text{2D}}$) were measured. The crystallite size ($L_{a}$) of the samples was estimated according to Equation (1).^[^ ^34^ ^]^

(1) $$\begin{array}{c} L_{a}\left[nm\right]=\left(2.4\;\times \;10^{-10}\right)\lambda_{l}^{4}{\left(\frac{I_{D}}{I_{G}}\right)}^{-1} \end{array}$$

#### 2.2.5 X‐ray Photoelectron Spectroscopy (XPS)

The chemical composition of the sample surfaces was analyzed by XPS at room temperature using a Nexsa G2 Surface Analysis System (ThermoFisher Scientific) with monochromatic Al Kα radiation. Data acquisition, processing, and analysis of the spectra were performed using the associated software Avantage Data System. KherveFitting software was used for deconvolution of the C 1s detail scans. The spectra were fitted by Gaussian–Lorentzian functions with 30% L/G Mix applying the included “Smart” background option, based on a Shirley background. Survey scans were carried out at a pass energy of 100 eV and an energy resolution of 1.0 eV, while high‐resolution spectra were recorded at a pass energy of 20 eV and a resolution of 0.1 eV. The relative atomic concentration was calculated from the integrated peak areas of major photoelectron spectral lines by subtracting the background. The C 1s line was used to calibrate the binding energy scale for the measurements, assuming a binding energy of 284.8 eV for C—C bonds. Hydrogen is omitted in the calculation of the surface composition. For each sample, two measurements with a spot size of 200 μm were conducted. C 1s peak fitting was carried out in accordance with parameters provided by Biesinger et al.^[^ ^35^ ^]^ and details of the peak deconvolution are provided in Table S2, Supporting Information.

### 2.3 Tribological Testing

The friction behavior of the LIG‐patterned surfaces was investigated in a linear reciprocating ball‐on‐plate configuration on a Universal‐Micro‐Tribometer UMT‐2 (Bruker Nano Surfaces Division) using 100Cr6 steel balls with a diameter of 6 mm (hardness HCR 60/62) as counterparts. The steel balls were cleaned with acetone prior to testing. The tribological tests were carried out under laboratory conditions (22 ± 2 °C and 30% RH). The test parameters were set to a normal load of 1 N, a constant linear velocity of 0.5 mm s^−1^ for a single stroke in the initial measurement, and up to 100 cycles in the reciprocating measurements with a stroke length of 8 mm. Each test was repeated at least twice per sample and carried out on three different samples for each pattern. Mean coefficients of friction were calculated after overcoming the static friction in single‐stroke measurements and in the steady‐state regime of reciprocating measurements reached after ≈20 cycles. The resulting COF evolution is plotted for the forward and backward strokes during the sliding movement, the turning points where the velocity becomes zero were excluded. The test setup is illustrated in Figure S1, Supporting Information, and a schematic representation of the contact condition between the counterpart and the sample is presented in Figure S2, Supporting Information.

## 3 Results and Discussion

### 3.1 Formation and Characterization of LIG Patterns

Polyimide was chosen as a substrate to fabricate samples for investigation of the tribological properties of patterned LIG structures, as it is a well‐established precursor material.^[^ ^36^ ^]^ A commercially available PI adhesive tape was applied on top of glass microscopy slides, as they provide stable support in the tribological test setup and allow for convenient sample handling. The LIG patterns were designed in 11 × 11 mm‐sized squares appropriate for the reciprocating ball‐on‐plate test setup. Four differently sized line patterns and a filled square were scribed.

Those structures were selected as different line patterns have already been explored as suitable textures to manipulate the tribological properties of a surface^[^ ^37^, ^38^, ^39^ ^]^ and are eligible for both the used texture fabrication process, and the measurement setup applied for tribological testing. To fabricate lines with widths of 200, 100, and 50 μm multiple laser passes were necessary to fill the relevant area. The distance between the individual passes was set to 15 μm. The 30 μm lines were scribed in a single pass and represent the smallest obtainable line width, which could be scribed using a UV laser with a beam size of 2 μm and the selected laser parameter. As there is no overlap between subsequent laser passes in the 30 μm pattern, the lower overall energy input (lower laser fluence *H*, defined as laser power per unit area^[^ ^1^ ^]^) may affect LIG formation. Therefore, possible implications on the characteristics of the 30 μm lines and potential distinctions to the other patterns have to be considered. In general, the difference between the beam size and the size of the scribed lines is a result of the area affected by local heating due to the laser pass, typically referred to as the heat affected‐zone (HAZ). Therefore, the resulting area of LIG conversion is always larger than the irradiated area.^[^ ^40^ ^]^ This effect was taken into consideration for the design step of the patterns to achieve the desired line width and spacing between individual lines. Furthermore, the surface morphology and composition of the LIG is determined by the laser parameters.^[^ ^40^, ^41^ ^]^ Thus, in an initial step, different laser parameters were evaluated. For this purpose, preliminary 100 μm line patterns were fabricated, where the influence of the laser parameters on the dimensions and defect‐free appearance was evaluated by optical microscopy, and the impact on the electrical resistance of the lines was measured with a multimeter. Further details of this parameter evaluation are displayed in Figure S3, Supporting Information. To limit influences from LIG formation, such as changes in morphology and effects on the mechanical properties, which in turn could affect the tribological properties, the selection of a single set of scribing parameters for the fabrication of all patterns was deemed appropriate. A parameter set resulting in a uniform graphene structure was selected for all subsequently assessed samples.

LIG formation is defined by its photochemical (bond cleavage and subsequent removal of functional groups that are recombined into volatiles) and photothermal (rearrangement of aromatic compounds and other carbon structures from sp^3^ to sp^2^ hybridized graphenic structures) contributions.^[^ ^42^ ^]^ Both reactions occur simultaneously but a dependence on the type of laser used and set laser fluence was reported.^[^ ^15^, ^42^ ^]^ Fiodorov and coworkers^[^ ^43^ ^]^ investigated how different wavelengths affect the conversion and showed that they also influence the photochemical versus photothermal contributions. The photon energy of infrared radiation is too low to directly break the chemical bonds in polyimide; therefore, the indirect heating effects are dominating. In contrast, the photon energy of the ultraviolet laser is high enough to directly break the bonds (the C—C bond energy is approximately equal to the 355 nm photon energy), which translates to a dominating photochemical effect.

Furthermore, a favored photochemical effect at low laser fluence and a favored photothermal effect at high laser fluence was discussed by Shin and coworkers.^[^ ^44^ ^]^ At low laser fluence, the threshold for LIG formation is not reached, and no consistent and continuous lines can be scribed. Under low fluence, only simple ablation of the material is observed, as commonly used in laser engraving. An increase in the laser fluence leads to the formation of thin carbonized sheets, followed by higher porosity due to the outgassing of oxygen and nitrogen. Subsequently, a transition from the porous graphene to outbreaking fibers occurs, which ultimately results in the destruction of the polyimide substrate at too high fluence.^[^ ^6^, ^40^ ^]^

**Figure 2:**
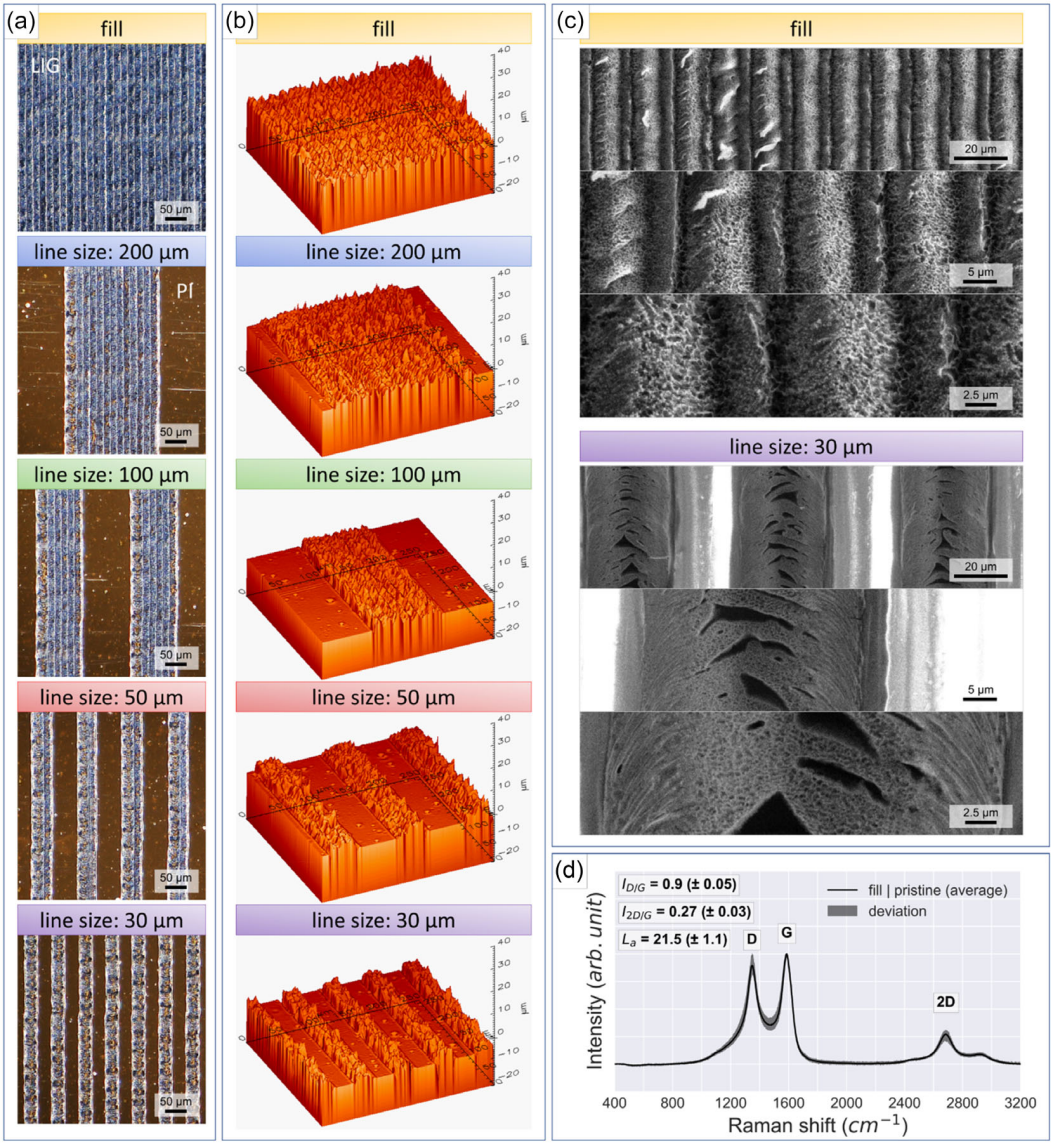
a) Optical microscopy images of the various UV‐LIG patterns on polyimide tape. b) 3D surface topographies obtained with confocal microscopy of 250 × 250 μm sized segments of the samples (fill and the individual line sizes). c) SEM images obtained with increasing magnification (top to bottom) of the LIG surfaces displaying the differences in LIG formation of the filled and single line (30 μm) sample, respectively. d) Raman spectra of the filled sample, which was averaged from six individual positions on three different samples. The intensity and shapes of the detected peaks are attributed to the defective graphene material characteristics of LIG.^[^ ^50^ ^]^

The line patterns obtained with a power of 15% and a scan speed of 55 mm s^−1^ were investigated by optical microscopy (**Figure** **2**a), evidencing that the individual lines were formed in a uniform and continuous manner. Conductivity measurements using a multimeter confirmed that the lines were not discontinued within the pattern, and an electrical resistance of ≈250 Ω was measured for the 11 × 11 mm filled squares. Depending on the laser settings, high fluence can also lead to vaporization of the PI film, which causes the distribution of finely pulverized particles on the surface.^[^ ^45^ ^]^ With the selected parameters, no particles were detected on the PI surface in between the scribed lines and the LIG is restricted solely to the irradiated areas.

Confocal microscopy images (Figure 2b) reveal that the laser‐induced graphene protrudes from the PI substrate, which lowers the number of contact points and allows for the counterpart to slide against the graphenic textures. SEM imaging (Figure 2c) shows the difference in morphologies of the filled LIG surfaced obtained by scribing multiple adjacent lines with a distance of 15 μm and a single scribed line. The raised feature of a single line does not exhibit a rectangular shape but more of a curved shape, which is higher at the edges compared to the center. Similar effects were previously discussed by Luo et al.^[^ ^6^ ^]^ and attributed to the non‐uniform distribution of the intensity of the laser beam over its diameter.

The obtained Raman spectra of the LIG structures (Figure 2d) feature three prominent peaks: the D peak, which is known as the disorder band and is a characteristic of the defective graphite structure at ≈1350 cm^−1^; the G peak, the primary mode in graphene and graphite, which is related to sp^2^ carbon lattice stretching vibrations indicating graphitic carbon at ≈1580 cm^−1^; and the 2D peak (or G’), caused by two phonon vibrational jumps in carbon atoms with anti‐directional momentum at ≈2700 cm^−1^.^[^ ^46^, ^47^ ^]^ The intensity ratio between the D band and the G band and the 2D band and the G band gives insight into the degree of graphitization of carbon materials. A higher $I_{D}/I_{G}$ ratio is indicative of lower quality LIG as the higher defect concentration increases the D peak intensity. Simultaneously, the 2D peak intensity is decreased by a higher defect concentration, and therefore, a lower $I_{\text{2D}}/I_{G}\;$ ratio characterizes LIG with a lower quality.^[^ ^9^, ^48^ ^]^ Furthermore, the $I_{\text{2D}}/I_{G}\;$ ratio is useful to assess the number of graphene layers and stacking order.^[^ ^46^, ^49^ ^]^ The measured $I_{D}/I_{G}$ ratio of 089 $I_{\text{2D}}/I_{G}\;$ ratio of 0.27 are in the range of various examples reported in literature, especially for LIG scribed using a UV laser source.^[^ ^43^, ^45^, ^50^, ^51^ ^]^ The comparably high degree of defects in the UV‐LIG can be linked to the occurrence of laser‐induced pyrolysis, which introduces disorder into the graphene lattice.^[^ ^49^ ^]^

### 3.2 Tribology of LIG‐Patterned Surfaces

**Figure 3:**
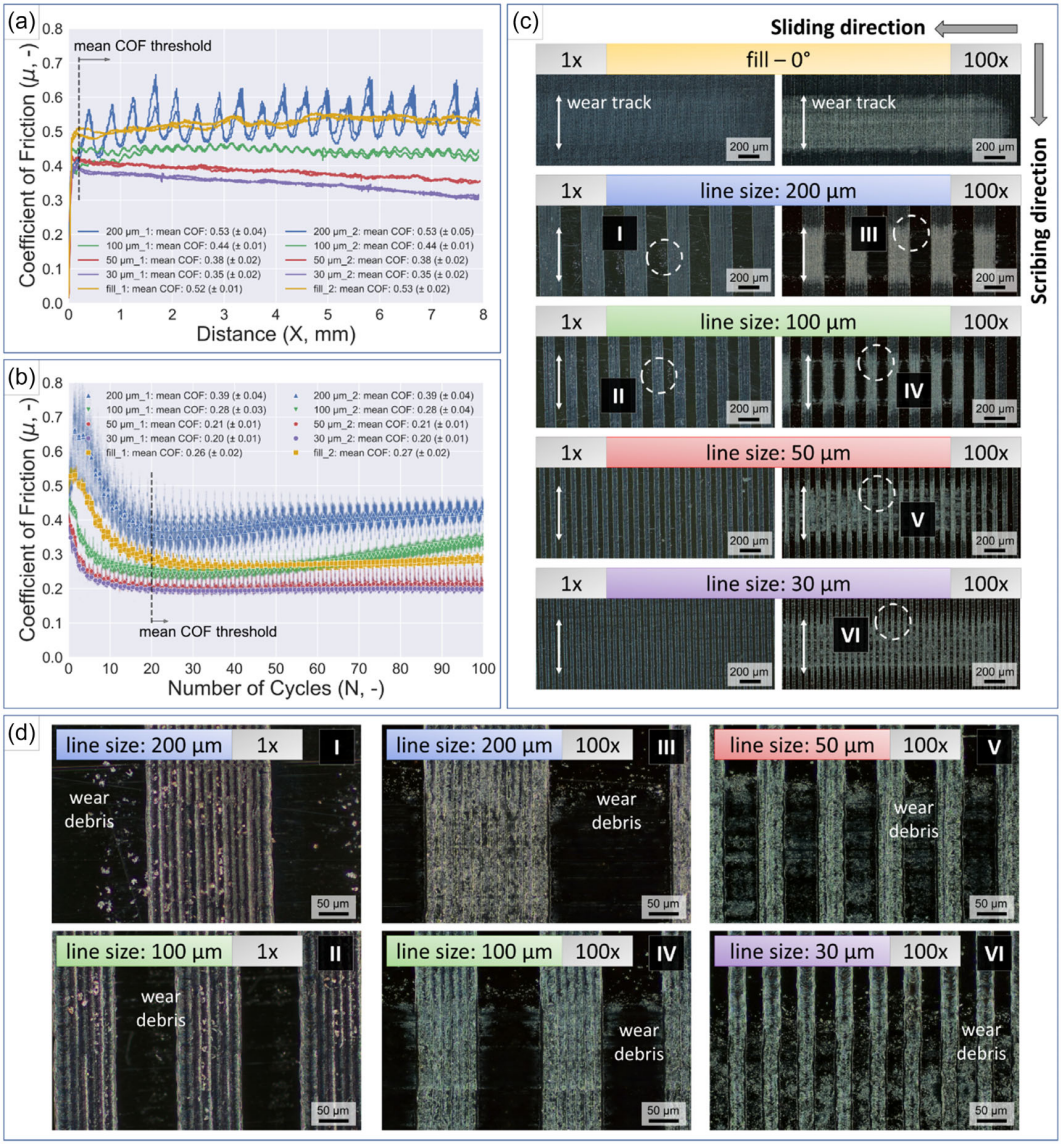
Comparison of the COF evolution of patterned and filled LIG samples from a) single‐cycle ball‐on‐plate measurements (8 mm sliding distance) and b) measured over a duration of 100 reciprocating cycles, which equates to a measurement duration of ≈30 min. Microscopy images of the resulting wear tracks after c) a single sliding cycle and 100 cycles. d) Close‐up images of the wear tracks to show the formed wear debris from areas highlighted in (c).

Tribological tests were conducted on the fully covered (fill) and patterned (200, 100, 50, and 30 μm lines) LIG surfaces. **Figure** **3** displays the comparative results of linear reciprocating tribological testing for a single cycle and 100 cycles, where the steel ball counterpart, with a diameter of 6 mm, moves over a sliding distance of 8 mm at a constant velocity of 0.5 mm s^−1^ and a normal load of 1 N on the LIG‐patterned PI samples. The sliding direction of the counterpart was perpendicular to the inscribed lines. Figure 3a displays the evolution of the coefficient of friction over a single stroke, the corresponding microscopy images of the resulting wear tracks are shown in Figure 3c. Friction curves from repeated measurements are available in Figure S4, Supporting Information. The wear tracks show little indication of wear of the LIG surfaces when the counterpart travels over the LIG structures only a single time. A small amount of wear debris, displayed as loose particles, is detectable on the laser‐scribed lines and in the spaces between them. Microscopy images of the wear debris of the 200 μm lines and 100 μm lines that were obtained with a higher magnification are shown in Figure 3d, position I and position II, respectively.

The friction curves exhibit a different evolution for the filled LIG surface compared to the surfaces with varying line sizes. The highest friction values were measured for the filled samples, with a mean COF of 0.53 (±0.02), and the 200 μm lines, with a mean COF of 0.53 (±0.04). The mean COF values were averaged in the dynamic regime after overcoming static friction. The distinct periodicity displayed by the 200 μm lines friction curve can be attributed to the comparably large spacing between the lines in relation to the contact area of the steel ball (schematically displayed in Figure S2, Supporting Information), which leads to an up and down movement of the steel ball as it passes each individual structure and gap respectively. This behavior manifests as a large oscillation amplitude of the COF, which has also been observed by other authors.^[^ ^52^, ^53^ ^]^ The friction curve of the 100 μm lines also features slight oscillation. Still, it is significantly less pronounced (i.e., smaller amplitude) compared with the larger 200 μm lines, as the steel ball is unable to descend and ascend as much into the gaps of the protruding LIG line pattern. The mean COF decreased to 0.45 (±0.01). Oscillation gets even less distinguishable when the line size is scaled down in samples with 50 and 30 μm lines. For these line sizes, which are very small compared with the diameter of the steel ball counterpart, the ball slides on top of the LIG lines without descending into the gaps between them. The mean COF decreases further to 0.38 (±0.02) for the 50 μm lines and to 0.35 (±0.02) for the 30 μm lines. Sung and coworkers^[^ ^52^ ^]^ studied the effect of surface topography on the frictional behavior at the micro‐ and nanoscale in 2003. They identified a relationship between the coefficient of friction and the geometric ratio (contact angle between the spherical counterpart and the microscopic groove structures), which can lead to a reduction of the coefficient of friction when the counterpart passes the grooved area compared to the flat region. A possible explanation of this occurring decrease in friction is a reduction of the true contact area in patterned surfaces.^[^ ^54^ ^]^ However, conclusions have to be drawn with caution as the understanding of true contact area in tribology is a challenging topic.^[^ ^55^ ^]^ This correlation between friction and surface patterns was also studied for patterned surfaces fabricated with laser surface texturing (LST), and a friction reduction for different line spacings was identified.^[^ ^54^, ^56^ ^]^ More factors that affect the tribological behavior of structured surfaces include hardness, plastic deformation, surface chemistry, adhesion, load contribution, and dispersion.^[^ ^57^, ^58^ ^]^

Subsequently, the tribological measurements were extended to prolonged testing durations of 100 cycles (50 forward and 50 backward strokes) of the linear reciprocating sliding motion. All test parameters were equal to those of the previously shown single‐stroke measurements, conducted over a length of 8 mm perpendicular to the inscribed lines. The evolution of the coefficient of friction over the entire measurement duration is displayed in Figure 3b; the corresponding microscopy images of the wear tracks, formed by the repeated sliding motion of the steel ball, are shown in Figure 3c. In contrast to the single cycles, a clearly pronounced wear track can be recognized for all samples. The recorded friction curves exhibit a running‐in period where the friction coefficients steadily decline from an initial higher value within approximately the first 20 cycles. This behavior can be explained by the different stages of the evolution of wear. When two pristine surfaces first come into contact in relative sliding motion, running‐in is defined as a complex interaction between friction, plastic deformation, and wear at the asperity level, which determines the resulting surface roughness, friction coefficient, and wear rate when a steady state is reached.^[^ ^59^ ^]^ Possible explanations for the decreasing friction coefficient include the gradual wear of the patterns, which reduces the surface roughness of the LIG line surfaces,^[^ ^56^ ^]^ the orientation of graphene planes (friction‐induced self‐orientation) that benefits the friction behavior,^[^ ^60^ ^]^ or a tribochemistry‐induced surface change.^[^ ^61^ ^]^ At the macroscopic scale, the tribological performance of graphenic materials is typically influenced by moisture and temperature. Therefore, a contributing effect of humidity on the evolution of friction during these longer durations is possible, as ambient conditions have been previously reported to affect tribological tests.^[^ ^62^ ^]^ A comparable running‐in behavior is observed for both the patterned and the filled LIG samples. However, the transition into the steady‐state appears to proceed quicker with decreasing line sizes. In the steady‐state, a more stable COF evolution is detected, which decreases with reducing line sizes. This evolution follows the previously discussed relationship in single‐stroke measurements, in which this correlation was attributed to the reduction of true contact. Additional explanations for the friction‐reducing effect of patterned surfaces include the ability of surface textures to trap and remove wear debris from the sliding interface.^[^ ^57^ ^]^ Hence, plowing is reduced. Furthermore, the textures can act as lubricant reservoir for solid lubricants.^[^ ^63^, ^64^ ^]^ The measured mean COF values in the steady‐state are as follows: 200 μm lines 0.39 (±0.04), 100 μm lines 0.28 (±0.03), 50 μm lines 0.21 (±0.01), 30 μm lines 0.20 (±0.01), and fill 0.26 (±0.02).

Assessment of the wear tracks after sliding shows a noticeable difference between the distribution of wear particles in the grooves between the LIG structures for the different line sizes. The wear tracks of the 200 μm lines features only a limited amount of remaining wear debris on the polyimide surface between the LIG‐patterned lines, accumulated mostly on the edge of the tracks (close‐up view, Figure 3d, position III).

The number of accumulated particles increases for the 100 μm‐lines and the particles still are mostly located on the edge of the wear track (Figure 3d, position IV). With a further decrease in line sizes (50 μm lines), a significant increase in the number of trapped particles is visible and the particles are much more evenly distributed over the entire width of the track (Figure 3d, position V). For the smallest evaluated 30 μm lines a very even distribution of the particles across the wear track can be observed, which suggests a correlation between line size and particle trapping ability. These different capabilities to capture wear particles might contribute to the different mean COF, which are reached in the steady‐state due to the abovementioned positive effect of reduced plowing or lubricant reservoir for solid lubricants. Figure S5, Supporting Information, shows microscopy images of the transfer film formed on the counterparts. The transferred LIG is mostly visible at the areas corresponding to the edge of the wear track and most pronounced for the 200 μm lines.

**Figure 4:**
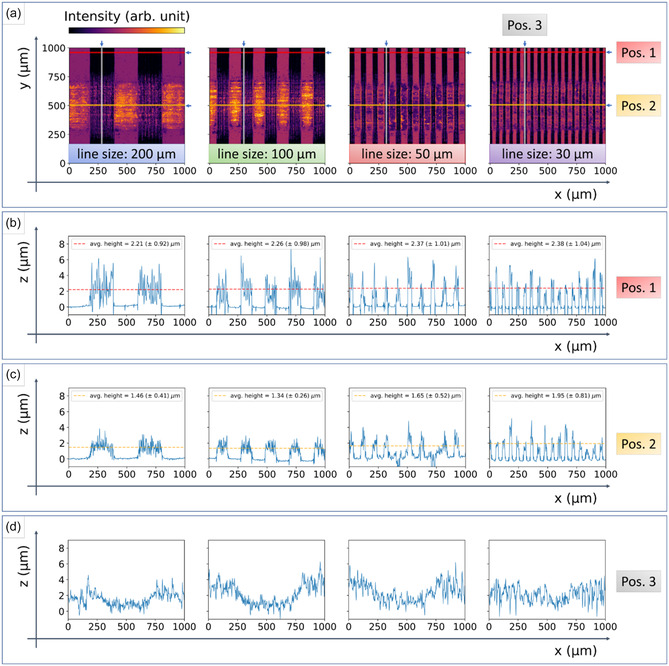
a) The extracted surface topographies of the various line patterns, sized 1 × 1 mm, are displayed in a color map with values related to the measured intensities. The different positions of the extracted height profiles are labeled and indicated with marked lines. b) Horizontal line profiles of an unworn area of the line patterns with calculated mean height values. c) Horizontal line profiles from the middle of the wear track after 100 sliding cycles. d) Vertical line profiles that show the groove formed by LIG wear caused by the repeated sliding motion of the steel ball counterpart.

To further investigate the effect of the tribological testing on the LIG‐patterned surfaces, confocal micrographs were taken to measure their surface topography and explore the friction‐induced changes. **Figure** **4** provides the obtained intensity images (a) and extracted height information (b, c, and d) from the 3D topographies. The height profile (Figure 4b) was extracted from the unworn area outside of the wear track and therefore corresponds to the pristine LIG surface. In comparison, Figure 4c displays the height profile extracted from the center of the wear track. Mean height values were calculated for the individual height profiles but should only be considered in a comparative manner, as the black surface of LIG is challenging for optical characterization techniques, resulting in measurement uncertainties stemming from, e.g., spikes and non‐measured points.^[^ ^65^ ^]^ The measured LIG height in the unworn area averages 2.31 (±1.0) μm with individual tips exceeding 6 μm. The height profiles extracted from the center of the wear track show a significant decrease for all samples compared to the unworn height profiles, with mean a LIG height of 1.60 (±0.5) μm. This finding supports the hypothesis of a wear mechanism, where the gradual wear of the LIG patterns causes a reduction of the surface roughness, which contributes to the reduction of the COF during running‐in. The smoothening of the tips and asperities is also reflected in the extracted vertical profile shown in Figure 4d.

Another interesting aspect that is revealed in Figure 4a is the worn PI tape (recognizable by the orange color due to higher intensity) in the wear tracks between the 200 and 100 μm lines. This can be traced back to abrasion caused by the relatively hard LIG wear debris, compared with the PI tape. In contrast, as the counterpart slides on top of the LIG structures for both 50 and 30 μm lines, not coming into contact with the PI gaps, no significant wear of the PI tape is detected. The latter finding has an additional influence on the evolution of friction.

**Figure 5:**
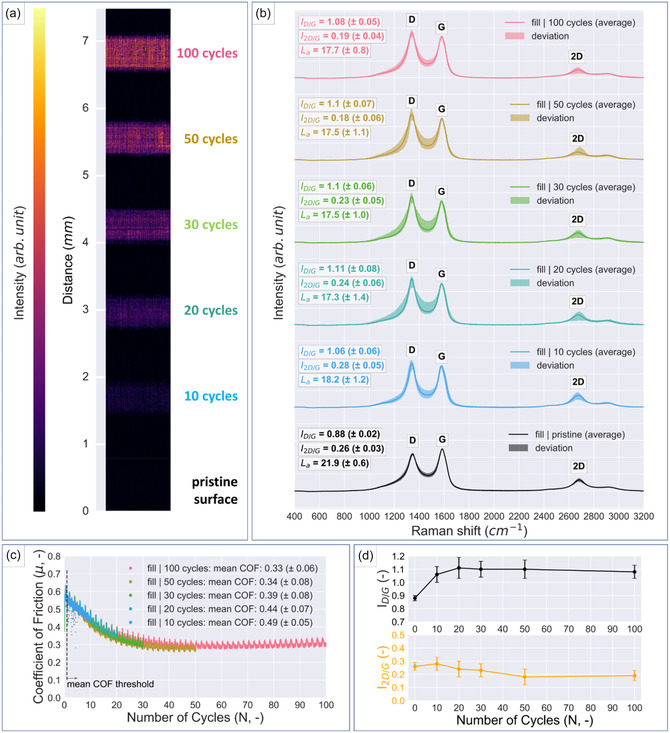
a) Confocal microscopy images of the wear tracks on a filled LIG sample formed by reciprocating linear sliding motion for a decreasing number of cycles (top to bottom) including the pristine LIG surface as a reference. b) Corresponding average Raman spectra and deviation between the repeat measurements obtained from each individual wear track. c) Comparison of the COF evolutions of the varied measurement durations of the filled LIG sample. d) Evolution of the $I_{D}/I_{G}$ and $I_{\text{2D}}/I_{G}$ ratios of the average Raman spectra as reported in (b).

To explore the effect of the tribological stress on LIG and its evolution of friction and wear, additional friction tests were carried out on a filled sample and stopped after a certain number of linear sliding cycles (100, 50, 30, 20, and 10 cycles, respectively). The formed wear tracks were evaluated by means of confocal microscopy, Raman spectroscopy, and XPS to study the running‐in behavior of LIG. The corresponding friction curves (**Figure** **5**c) show that the evolution of the COF approaches a steady‐state value of ≈0.3, which is reached after around 35 cycles. Figure 5a displays confocal images of the formed wear tracks and the change in detected intensity (represented by the change in color) correlates to the degree of wear.

The respective Raman spectra are shown in Figure 5b, including the average spectra, scattering of the repeat measurements and the calculated $I_{D}/I_{G}$ and $I_{\text{2D}}/I_{G}\;$ ratios. The shift in the $I_{D}/I_{G}\;$ and $I_{\text{2D}}/I_{G}\;$ ratios over the number of measured cycles is presented in Figure 5d. The spectra were normalized to the G peak to visualize the changes in D peak, over the evolution of the tribological tests. An $I_{D}/I_{G}\;$ ratio of 0.88 (±0.02) was measured for the pristine LIG surface, which increased to 1.08 (±0.05) after 100 cycles. The increase in the $I_{D}/I_{G}\;$ ratio with an increasing number of sliding cycles signifies a more defective carbon structure. Due to the mechanism of LIG formation, where the LIG essentially grows from the PI substrate, the morphology of the LIG varies over its height profile.^[^ ^45^ ^]^ The changes in the Raman spectra indicate that sliding against the counterpart wears down the top layers of LIG, which are richer in graphene and reveal the underlying, more defective, morphologies. The significant reduction in COF during the running‐in phase can be correlated when comparing Figure 5c and the $I_{D}/I_{G}\;$ ratio in Figure 5d. In addition to the decrease in surface roughness resulting from the abrasion of LIG roughness tips, other factors likely contribute to this significant decrease in COF and change in the Raman spectra. Tribological stress is a known cause of surface layer formation, resulting in a different microstructure compared with the pristine material. Therefore, contributing factors to this decrease in COF from friction‐induced changes include the previously mentioned orientation of graphene planes and the ability of graphenic materials to self‐lubricate due to their film‐forming abilities.^[^ ^27^ ^]^ In addition, the scattering of the single Raman spectra is most prominent on wear tracks after 20, 30, and 50 cycles, explained by the progression of the running‐in process, where both worn and unworn areas occur simultaneously. As the sliding duration increases further (100 cycles), the surface is mostly worn, which leads to reduced scattering between the individual measurements on the wear track. The apparent broadening and overlap between the D band and the G band is an indication of a certain degree of amorphous carbon in contrast to the distinct D and the G bands of LIG. The reduction of intensity of the 2D band further supports this revealed changed LIG morphology in the wear tracks due to frictional load.

Established $I_{\text{2D}}/I_{G}\;$ ratios of single‐, double‐, triple‐, and multilayer graphene are in the range of > 1.60, 0.80, 0.30, and 0.07, respectively.^[^ ^49^ ^]^ An $I_{\text{2D}}/I_{G}\;$ ratio of 0.26 (±0.03) was measured on the pristine LIG surface, which indicates the occurrence of multilayer graphene. Furthermore, the $I_{\text{2D}}/I_{G}\;$ ratios decreased to 0.19 (±0.04) on the wear track formed by 100 sliding cycles, which is also an indicator of a more defective carbon structure.

The measured intensity of bands in Raman spectra can be used to estimate the crystallite size $L_{a}$ using Equation (1). Measured $L_{a}$ values decrease from 21.8 nm of the pristine LIG to 17.8 nm after 100 sliding cycles, implying that the tribological process could partially destroy the crystalline domains.^[^ ^66^, ^67^ ^]^ The observed pristine value aligns with similar findings reported in literature for LIG from polyimide.^[^ ^68^, ^69^ ^]^ Typically, $L_{a}$ values higher than 20 nm indicate a graphene material consisting of relatively large domains. In comparison, $L_{a}$ values lower than 20 nm are representative of relatively small and potentially numerous crystalline domains.^[^ ^49^ ^]^

Low‐resolution survey scans and high‐resolution detail scans of the O 1s and C 1s regions of the pristine LIG surface, and the formed wear tracks are provided in Figure S6, Supporting Information. The detected spectra of the pristine LIG surface show the characteristic dominating C 1s peak (84.0 (±0.8) at%, consisting mainly of sp^2^‐carbon),^[^ ^1^ ^]^ a significantly less pronounced O 1s peak (12.0 (±1.4) at%, related to the ambient atmosphere present during the laser‐induced pyrolysis)^[^ ^70^ ^]^ and small contributions of the N 1s peak (4.0 (±0.6) at%, attributed to the PI precursor material).^[^ ^1^ ^]^ In comparison, the spectra obtained from the worn tracks show a slightly increased C 1s signal and lowered O 1s and C 1s signals (Table S1, Supporting Information), which corroborates the differences in LIG morphology in the wear tracks. C 1s deconvolution reveals comparable LIG binding states to those reported by Zhang and coworkers.^[^ ^71^ ^]^ However, the variations between the measured XPS spectra are too minor to draw definitive conclusions, as the references for accurately deconvolving laser‐induced graphene spectra are limited.

## 4 Conclusions

In this study, we investigated the fabrication of selected patterns with varying line widths and spacings on polyimide tape using a single‐step irradiation with a UV laser source. In contrast to commonly applied IR lasers for LIG formation, UV laser sources benefit from a smaller spot size, allowing for scribing with a higher spatial resolution. Here, we were able to scribe continuous lines with a minimal resolution (line width) of 30 μm. However, compared to LIG fabricated on PI tape using an IR laser source, UV‐lasing yields a more defective LIG, as it was identified by Raman spectroscopy. This partial trade‐off between spatial resolution and LIG quality has to be taken into consideration for the intended applications. The used laser scanning system allows for control over the scribing parameters, including speed and directions of scribing, which enables a simple and flexible production of a wide range of patterns. Therefore, laser scribing provides a convenient approach to introduce surface patterns to influence the tribological properties. As laser scribing can be applied to more and more materials, opportunities arise to introduce surface textures onto various substrates in a convenient, fast, and maskless process.

Tribological testing in a linear reciprocating ball‐on‐plate setup showed that LIG exhibits an initial coefficient of friction (COF) of 0.52, which decrease significantly during running‐in to a mean value of 0.26. Optical microscopy, confocal microscopy, XPS, and Raman spectroscopy were used to investigate the running‐in process and wear mechanism. Confocal microscope measurements of the wear tracks at certain stages of running‐in revealed a gradual wear of the LIG. Thereby exposed LIG morphologies show an increase in $I_{D}/I_{G}$ ratios, a decrease in $I_{\text{2D}}/I_{G}$ ratios, and a reduction of the crystallite size $L_{a}$ of the respective Raman spectra, signifying a more defective carbon structure. A significant effect on the tribological behavior from introducing line patterns was established. The mean COF was highest for the 200 μm lines (0.39 in the steady‐state) and decreased for the smaller line sizes 100 μm lines, 50 μm lines, and 30 μm lines, respectively. The measured COF reached its lowest steady‐state value of 0.20 for the 30 μm lines. The wear mechanism of the line patterns was investigated, and a deviation between the distribution of wear debris corresponding to the particle trapping ability of the individual patterns was most prominent.

The underlying mechanisms responsible for the significant decrease in COF during the running‐in stage are not yet fully understood, as the tribological properties of a material always result from a superposition of multiple effects. An abrasive wear mechanism, which reduces the surface roughness of the LIG peaks and forms fine, loose wear debris that remains in the contact and possibly contributes a lubricating effect characteristic of graphene materials, is indicated. The influence of LIG formation (e.g., changes in morphology from different laser sources or scribing parameters) on friction behavior has not been explored. Therefore, to utilize the potential of LIG as a solid lubricant, uncovering these structure‐property relationships is crucial and could help to reduce friction even further compared with the results shown in this work.

Furthermore, a distinct delimitation of contributions from variations in LIG material characteristics and the effect of different patterns on the tribological properties is currently still limited. Thus, this would be a key direction for future research to enable the exploitation of patterning effects in governing the friction behavior and to make LIG eligible for its use in tribological applications.

## Supporting Information

Supporting Information is available from the Wiley Online Library or from the author.

## Conflict of Interest

The authors declare no conflict of interest.

## Supporting information

Supplementary Material

## Data Availability

The data that support the findings of this study are available from the corresponding author upon reasonable request.
