# Supplementary material for: Friction and Wear Behavior of Laser‐Induced Graphene Structures on Polyimide Films
Source: Small Sci. 2025 Oct 15;5(12):e202500335. doi: 10.1002/smsc.202500335 (PMC12697860; doi:10.1002/smsc.202500335)
Supplement: Supplementary file 1 — Supplementary Material [file SMSC-5-e202500335-s001.pdf]

Supporting Information

Friction and Wear Behavior of Laser-Induced Graphene Structures on Polyimide Films

Milena Gleirscher, Stefan Zeiler, Paola Parlanti, Christine Bandl, Verena Maier-Kiener, Francesco Greco, Sandra Schlögl**

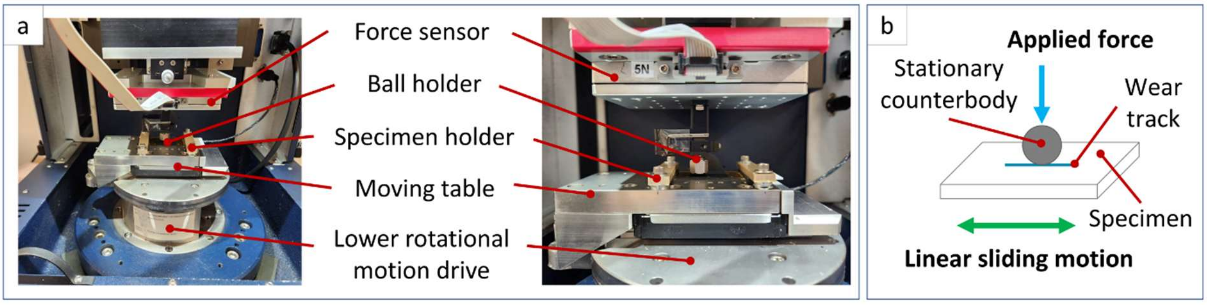

Figure S1: a) Illustration and b) schematic representation of the reciprocating ball-on-plate measurement setup on the UMT-2 tribometer.

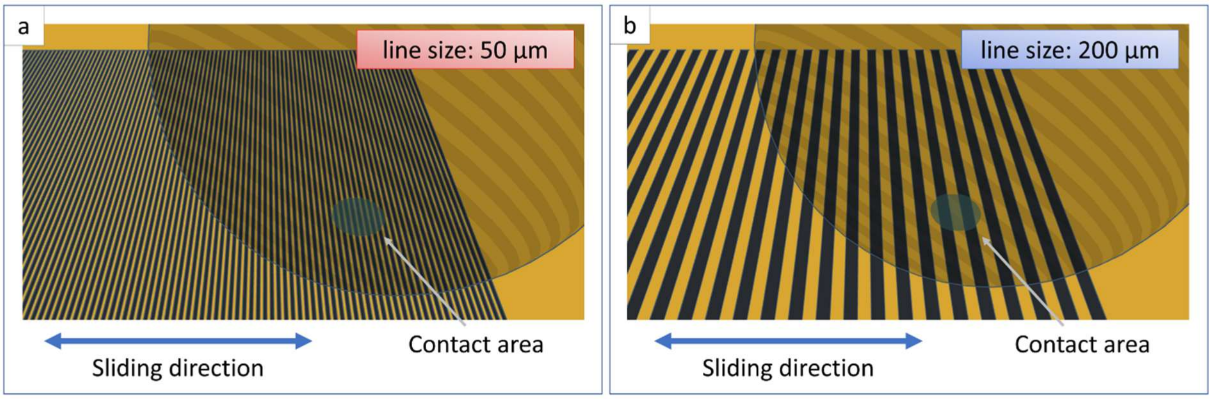

Figure S2: 3D representation of the size ratios between the contact of the line structures and the steel ball for line sizes of a) 50 µm and b) 200 µm.

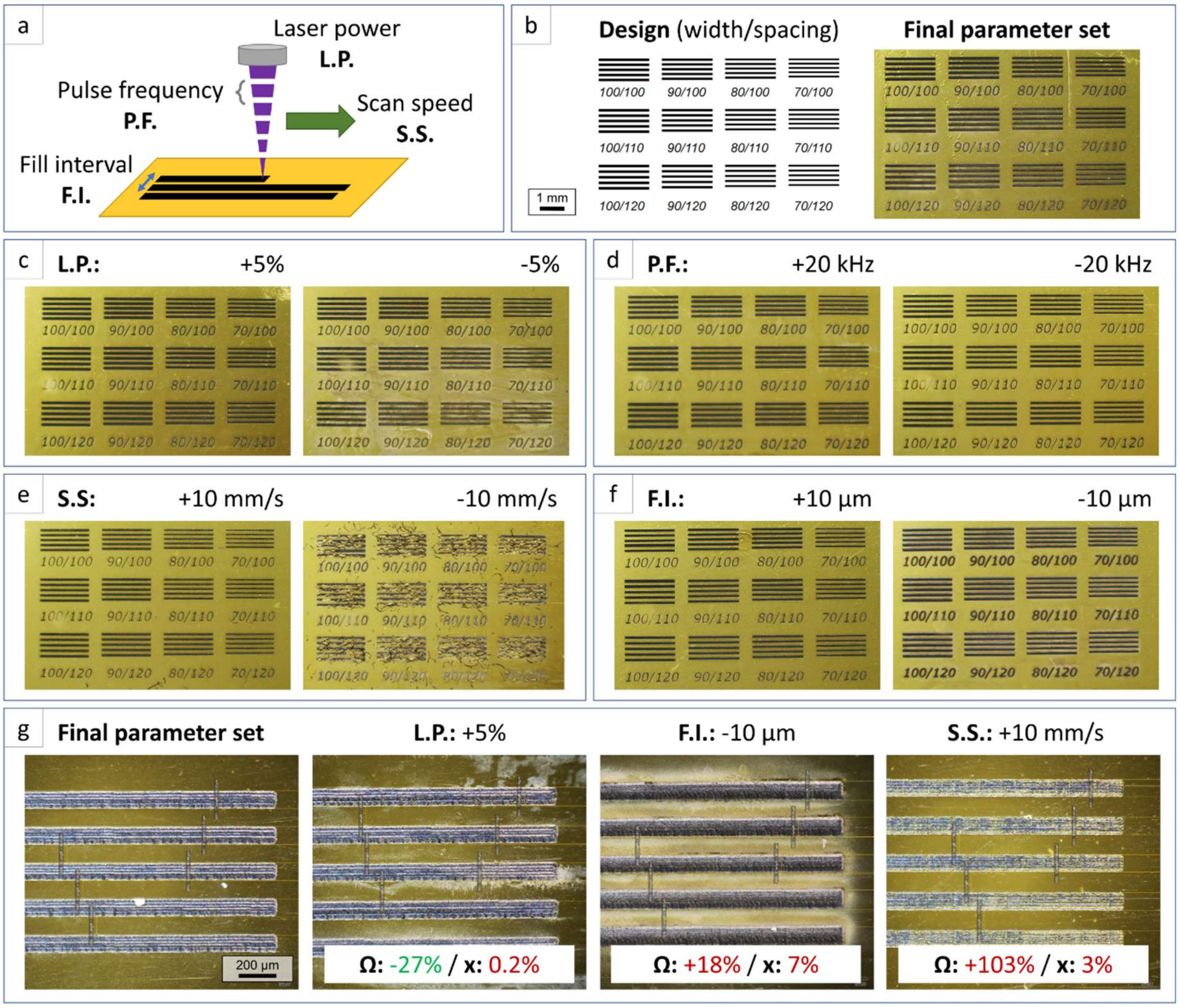

Figure S3: Preliminary tests for laser parameter selection. a) Schematic representation of adjustable scribing parameters. b) The utilized design and the final parameter set, which was used throughout the study. Altered parameters from the optimized set: c) laser power, d) pulse frequency, e) scan speed, and f) fill interval. g) Exemplary representation of the evaluation of the individual lines considering the occurrence of powdered LIG, the change in electrical conductivity (indicated by "Ω"), and the deviation from the line width (indicated by "x") as selection criteria. The scale bar displayed in b) is valid for images c) to f).

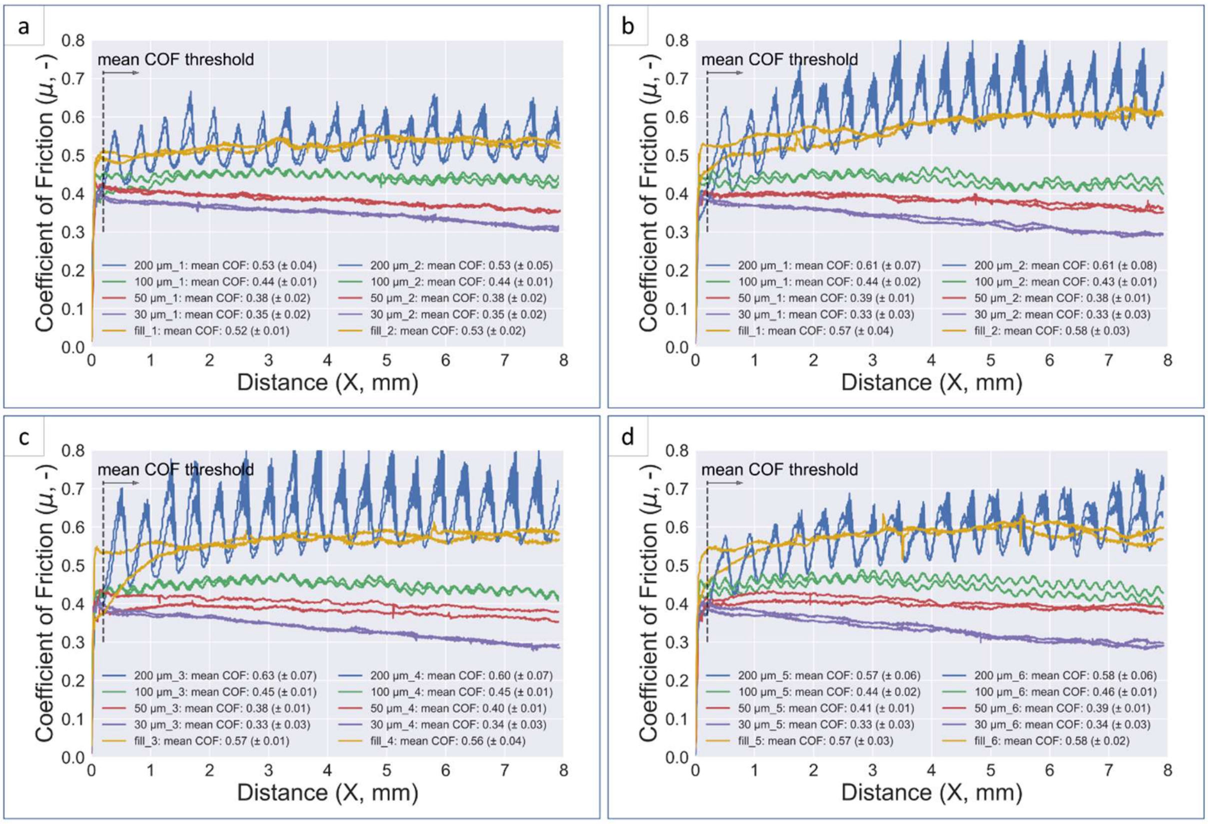

Figure S4: Comparison of the COF evolution of patterned and filled LIG samples from repeat measurements of single-cycle reciprocating ball-on-plate measurements with a sliding distance of 8 mm.

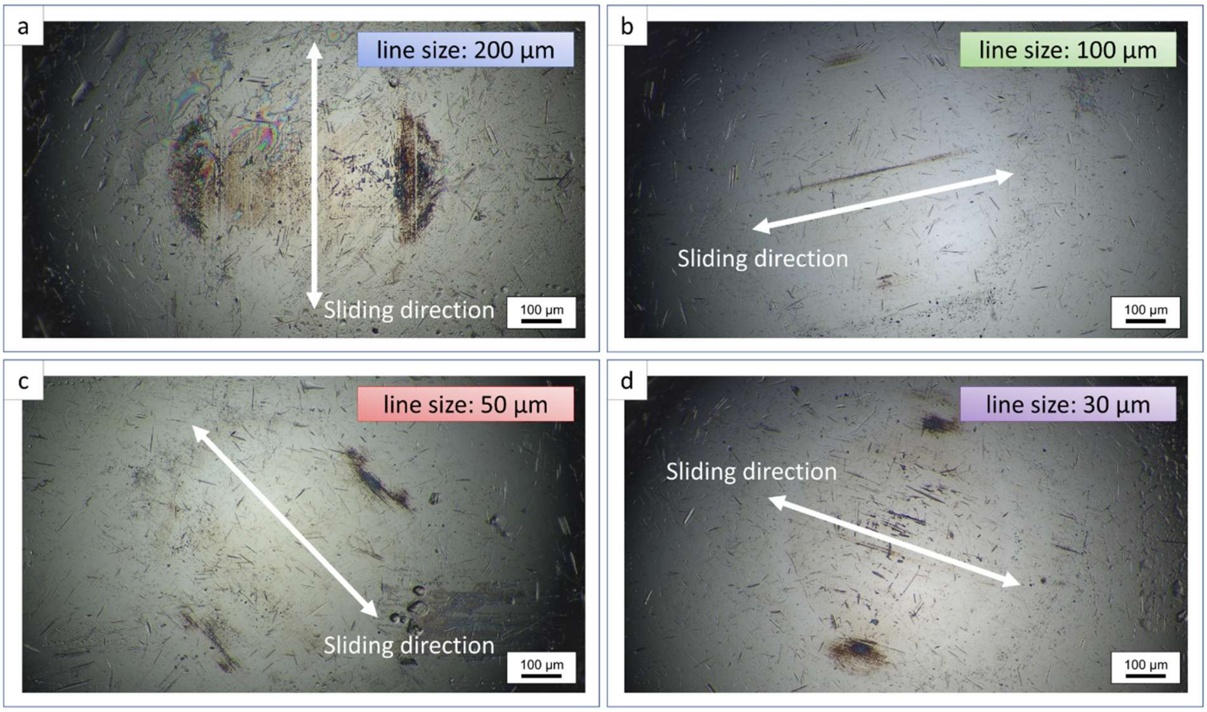

Figure S5: Microscopy images of the formed transfer film on the steel ball counterparts from reciprocating ball-on-plate sliding tests with a measurement duration of 100 cycle of samples a) 200 μm lines, b) 100 μm lines, c) 50 μm lines, and d) 30 μm lines.

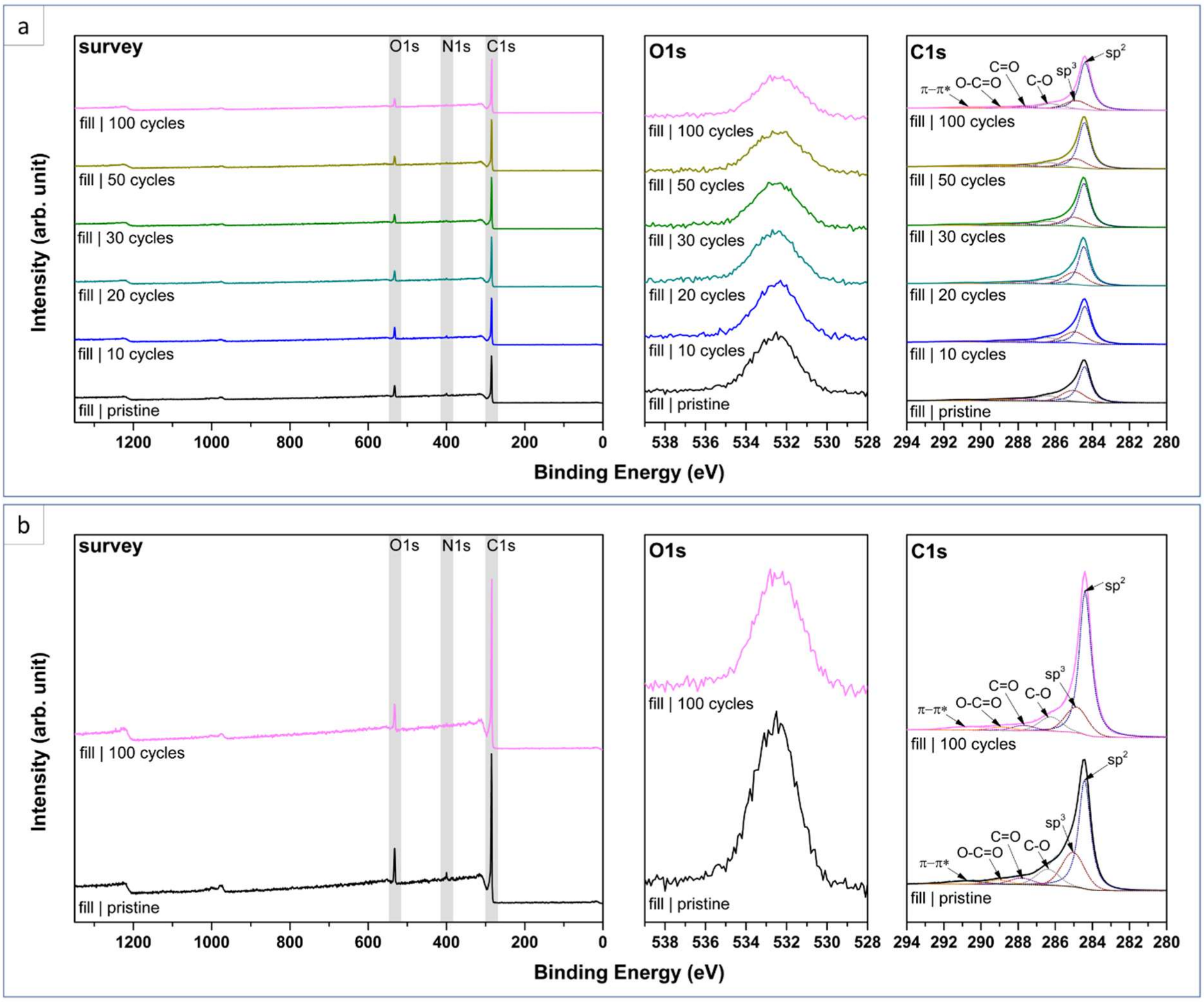

Figure S6: Comparison of XPS survey, O 1s, and C 1s detail scans obtained from the wear tracks of the filled LIG sample for a) a decreasing number of sliding cycles (top to bottom: 100 cycles to pristine LIG) and b) direct comparison between XPS spectra obtained after 100 cycles of reciprocating linear sliding motion (top) compared to the pristine surface (bottom).

Table S1: Overview of the elemental composition of the LIG surfaces obtained from the XPS survey scans on the wear tracks of the filled LIG sample and the pristine filled LIG surface.

Peak	Peak Energy (eV)	Composition (at%)					
		fill | pristine	fill | 10 cycles	fill | 20 cycles	fill | 30 cycles	fill | 50 cycles	fill | 100 cycles
C 1s	284.82 (± 0.20)	84.0 (± 0.8)	87.5 (± 0.1)	87.6 (± 0.5)	89.8 (± 1.0)	90.6 (± 0.6)	89.6 (± 1.9)
N 1s	400.02 (± 0.16)	4.0 (± 0.6)	2.3 (± 0.2)	2.8 (± 0.6)	1.6 (± 1.6)	1.9 (± 0.5)	2.2 (± 2.2)
O 1s	532.43 (± 0.12)	12.0 (± 1.4)	10.2 (± 0.1)	9.6 (± 1.0)	8.6 (± 0.6)	7.5 (± 0.1)	8.5 (± 0.1)

Table S2: Overview of peak fitting parameters of C 1s high-resolution detail scans displayed in Figure S6. The spectra were deconvoluted into six peaks assigned to sp^2-carbon, sp^3-carbon, C-O, C=O, O-C=O, and the π-π* shake-up satellite by applying the indicated position and FWHM constraints.

**ID**	**Label**			**Peak Energy (eV)**				**Position Constraint (eV)**
	C 1s	**fill | pristine**	**fill | 10 cycles**	**fill | 20 cycles**	**fill | 30 cycles**	**fill | 50 cycles**	**fill | 100 cycles**	
A	sp^2	284.42	284.41	284.46	284.45	284.43	284.40	284.5
B	sp^3	285.04	284.96	284.96	284.99	284.96	284.86	A + 0.5 (± 0.2)
C	C-O	286.40	286.40	286.40	286.36	286.33	286.25	A + 2.0 (± 0.3)
D	C=O	287.82	287.82	287.82	287.75	287.73	287.70	A + 3.5 (± 0.2)
E	O-C=O	289.03	288.82	288.93	289.05	289.08	288.91	A + 4.5 (± 0.3)
F	π- π*	290.88	290.71	290.78	290.92	290.84	290.70	A + 6.4 (± 0.1)

**ID**	**Label**			**FWHM (eV)**				**FWHM Constraint**
	C 1s	**fill | pristine**	**fill | 10 cycles**	**fill | 20 cycles**	**fill | 30 cycles**	**fill | 50 cycles**	**fill | 100 cycles**	
A	sp^2	0.75	0.76	0.73	0.78	0.76	0.75	0.3:0.8
B	sp^3	1.49	1.49	1.47	1.5	1.5	1.49	0.9:1.5
C	C-O	1.49	1.49	1.47	1.5	1.5	1.49	B*1
D	C=O	1.49	1.49	1.47	1.5	1.5	1.49	B*1
E	O-C=O	1.49	1.49	1.47	1.5	1.5	1.49	B*1
F	π- π*	2.46	2.58	2.51	2.54	2.68	2.69	2.7 (± 0.1)

**ID**	**Label**			**Concentration (%)**				**Fitting Function**
	C 1s	**fill | pristine**	**fill | 10 cycles**	**fill | 20 cycles**	**fill | 30 cycles**	**fill | 50 cycles**	**fill | 100 cycles**	
A	sp^2	51.5	54.1	52.1	60.7	61.3	62.6	LA
B	sp^3	25.3	25.0	26.6	18.8	18.7	17.5	GL
C	C-O	11.7	10.6	10.7	9.3	9.3	9.7	GL
D	C=O	4.8	3.8	4.3	4.0	3.8	3.6	GL
E	O-C=O	3.0	2.7	2.6	3.0	2.7	2.3	GL
F	π- π*	3.6	3.8	3.6	4.2	4.3	4.3	GL
